# The Hospital. Nursing Mirror

**Published:** 1898-06-04

**Authors:** 


### "The Hospital" Nursing Mirror.

Being the Nursing Section of "The Hospital."

[Contributions for this Section of "The Hospital" should be addressed to the Editor, The Hospital, 28 & 29, Southampton Street, Strand, London, W.C., and should have the word "Nursing" plainly written in left-hand top corner of the envelope.]

### News from the Nursing World.

### IMPROVEMENTS AT ST. BARTHOLOMEW'S.

The removal of Christ's Hospital to Horsham leaves a valuable site at the disposal of the governors. It is reported that negotiations are on foot with the authorities of St. Bartholomew's for the transfer of a portion of the buildings. It is well known that the residential college for the medical students is not altogether satisfactory, and that the hospital will be glad to acquire extensive and convenient premises for a nurses' home.

### FRENCH WOMEN IN TIMES OF WAR.

The ever-present possibility of war has spurred both the men and women of France to hold themselves in readiness. The women long ago organised an association called "The Union of the Women of France," which now possesses 175 buildings and 36,000 members. If war were declared it could equip 19 field hospitals and 1,900 beds, staffed with 1,200 certificated nurses. It is an object lesson for ourselves. Our army nursing service is by no means adequate, and except the Princess Christian's Army Nursing Reserve Corps, there is no provision for an outbreak of hostilities. It is sincerely to be hoped that peace may be preserved, but it is always well to be prepared for the worst. There is plenty of material, trained and willing nurses in abundance, but organisation takes time, and a nursing militia could be called out and rendered efficient much more quickly than would be the case with volunteers.

### NURSING AT HASLEMERE.

For some time Mr. Penfold and his sisters have been desirous of undertaking some work of public importance as a memorial of their late parents. The want of accommodation for two or three bad accidents drew their attention to the need of a cottage hospital for Haslemere and the parishes in the immediate neighbourhood. The result is that at the annual meeting of the nursing associating held recently a new freehold building for a cottage hospital and nurses' home on Courts Hill was formally handed over to three trustees to be used for this purpose. The hospital contains two wards (each accommodating two patients), six bedrooms, two sitting-rooms, kitchen, scullery, bath-room, &c. There is also a detached mortuary. The institution is still to be furnished, for which £94 has already been received, and the maintenance will depend upon public support. Two nurses have been employed by the association throughout the year, and a third for some weeks. After all expenses have been met there remains a credit balance of £11.

### ESSEX COUNTY COTTAGE NURSING ASSOCIATION.

The Essex County Cottage Nursing Association comes before its friends with an excellent record. At the annual meeting, over which the Lord Lieutenant of the County (Colonel Archer Houblon) presided, the ordinary report for 1897 was read, and an account was given of the way in which the Jubilee offering of £2,000 had been dealt with. The total income, including the £2,000 already mentioned, reached £2,744; £1,000 of this was invested in debenture stock, £710 was spent in buying the freehold of the nursing home at Harrow Green, and £265 in its maintenance. There is a balance in hand of £454. Arrangements have been made to build a lecture-hall, with bedrooms for probationers over it. When these are ready two rooms in the home will be given up to a *crêche*, which is much needed in the neighbourhood, and to which donations have already been promised. Since the formation of the association 52 women have been given a course of training, and there are now ten probationers receiving instruction.

### NURSING OUTDOOR PAUPERS.

An instructive discussion upon the nursing of outdoor paupers took place at a recent meeting of the Loughborough Board of Guardians. It arose out of the question of granting £20 a year to the District Nursing Association. From the board's point of view it resolved itself entirely into a commercial transaction as to whether it would be cheaper to engage a nurse of their own or pay the association to do the work. How it would have fared for the association had there not been voluntary district nurses employed in other parishes under the board, which might in due time claim grants in their turn, it is impossible to say; as it was, only nine members voted in favour of the grant and sixteen against it. In the meantime the sick paupers are terribly neglected, and Dr. Phelps drafted the following motion: "That the destitute sick of this town receiving anything but proper nursing attention, the board consider the advisability of remedying the evil."

### COTTAGE NURSING IN BERLIN.

The Home Nursing Society, a branch of the Berlin Frauer-Verein, has issued the report of its first year's work. The object of this society is to provide untrained attendants to supplement the work of the midwives and trained nurses. These attendants discharge the usual routine duties that usually fall upon the mothers in poor families, and so afford great assistance both to the sick woman and the trained nurse attending her. This attempt to solve the problem of providing the invalid with domestic help as well as with trained nursing is worthy of consideration. Germans are celebrated for method and economy, and this system leaves the highly-trained district nurse free from drudgery, whilst it confines the duties of the unskilled attendant to the kitchen and laundry, thus meeting the strongest plea put forward on behalf of instituting an inferior grade of cottage nurse. The objection to it is, of course, the expense in poor districts.

### DERBY AND DERBYSHIRE NURSING ASSOCIATION.

The thirty-third annual meeting of the Derby and Derbyshire Nursing Association was held recently. The past year has been one of progress and encouragement, the services of the nurses having been in greater demand than ever. A superintendent has been appointed to the district staff, which now numbers seven. In future all nurses who have been five years on the private staff will be given a bonus of five per cent. on their earnings. District nurses who have served as long will also have similar bonuses computed at the ordinary rate of charges. The arrangements for receiving nurses in quarantine have been greatly improved. The building has been lengthened, the roof raised, and the windows enlarged. The staff now consists of 45 engaged in private nursing, eight in district work, and 12 probationers. Their health has been fairly good; several have suffered from influenza, two from typhoid, and one from jaundice. The income for the year was £3,077, and the expenditure £2,649.

### NURSING IN THE "BRITANNIA" HOSPITAL.

It appears from an answer given by Mr. Austen Chamberlain, on behalf of the First Lord of the Admiralty, that in 1894 a trained nursing sister was appointed to superintend the ordinary hospital staff of the "Britannia" Hospital, and that another trained nurse was added in 1896. Both ladies have had long experience in civil hospitals, and were chosen on account of their fitness to fill their present positions.

### NURSING IN INDIA.

It is interesting to note from the third annual report of the Up-Country Nursing Association that the cost of placing a nurse in India is about £72. The local committee of the north-west provinces has severed its connection with the Ramsay Hospital, where there was no longer room for its nurses. It has established a nursing home fully furnished and possessing an adequate staff of servants, with results that fully justify the additional outlay. The financial position of this branch is better than it has ever been. A heavy debt has been discharged towards the home committee, and the year has closed with a very satisfactory credit balance. The Quetta branch has been closed as reported last year, Nurse Gray, who was invalided home, arriving in England in the March of last year. A Punjab branch has been established, which has its headquarters at Lahore, and of which Lady Young is president. Three nurses are already at work there.

### JUBILEE MEMORIAL AT SOUTHAMPTON.

The report of the sub-committee appointed to carry out the scheme for the erection of a nurses' home, as a memorial of the Queen's Diamond Jubilee, was read and adopted at a public meeting in Southampton Town Hall on the 20th inst. It stated that the terms for leasing a site, generously offered by the Right Hon. Evelyn Ashley, have been agreed to. Mr. Ashley, having approved the plans of the new home, it was decided to invite tenders for the building. The site immediately joins the Creamery, near the Palmerston Lodge, and the proposed home will have four bedrooms upstairs, with three sitting-rooms, a kitchen and scullery on the ground floor. It will be built of red brick with stone facings.

### PETERBOROUGH DISTRICT NURSING ASSOCIATION.

The annual meeting of the Peterborough District Nursing Association was held at the infirmary on May 5th, when Mr. T. H. Beeby took the chair. The Superintendent, after reading her report, remarked of the nurses under her that "they were trained nurses, appointed to act under doctor's orders, and should not do any other work," and maintained that doctors were the best judges as to whether the services of a trained nurse were necessary, or whether "a little temporary help from a charwoman would be more to the point." Miss Peter, the inspector of Q.V.J.N.I., gave a satisfactory report, and congratulated the nurses on their pretty new home. One hundred and ninety pounds a year is required to meet all expenses.

### A LEAFLET FOR ASYLUM NURSES.

The Rev. H. Hawkins, chaplain of Colney Hatch Asylum, whose interest in asylum workers is well known, has issued, through Messrs. Potter, Batter, and Davies, two short addresses, one of welcome and advice, and one of farewell, to nurses engaged in this arduous work. The leaflet is entitled, "The New Asylum Nurse," and is full of helpful suggestions and wise counsel.

### SHORT ITEMS.

Mrs. Phillips, the Nurses' Association, St. Leonards-on-Sea, desires us to state that this institution has no connection with the Nurses' Co-operation, St. Peter's Road.—The Lord Mayor of Manchester formally opened the nursing home at Carlton-on-Trent on the 26th ult., and promised to subscribe £5 5s. annually to its funds.—Mrs. Sarah Harrison's death at the age of 96 is worthy of note. She was child's nurse to General Gordon and his brother, and for the few days the Duchess of Kent and the Princess Victoria spent with the Gordons at Barnby Dun on their way from London to York she had the pleasure of waiting on her future sovereign.—Mrs. Wilkie, president of the Chirnside and District Nursing Association, has promised the use of Foulden House and grounds about the beginning of August for the garden fête and sale of work to be held in aid of the society's funds.—The recent private theatrical performance at the Grand Theatre, Cardiff, in aid of the local branch of Queen Victoria's Jubilee Institute for Nurses, resulted in the sum of £50 being handed over to the treasurer, Dr. Sheen.—Lady Crossman, of Cheswick, died on Monday night, the 23rd ult. She has recently returned from the Continent, where she had been in search of health. Lady Crossman was president of the Ladies' Queen's Jubilee Nursing Association at Berwick from its establishment until her death.—The *Gentlewoman* for May 28th contains a photograph of the group of Dublin nurses taken at the Countess of Cadogan's garden party.—A committee has been appointed by the Poole Guardians to take in hand the necessary alterations of the nurses' quarters in order to make them healthy and comfortable.—Rs. 2,000 are still needed to recompense the lady nurses for their loss by the fire at Modikhana. It is pleasant to note that an appeal for the amount appears in the *Times of India* from a Parsee subscriber to the fund.—Miss Margaret Astor Chanler, an American millionaire, is training for work as a Red Cross nurse.—Miss R. W. Rattray, of Dublin, has been appointed superintendent nurse of the Prescot Union Infirmary. The board of guardians having recently acquired the Holt House estate, the house upon it has been turned into a nurses' home, and a new infirmary has been built.

### Lecture to Nurses.

### ON PREPARATION FOR AND AFTER TREATMENT OF CASES OF ABDOMINAL SECTION.

By E. Stanmore Bishop, F.R.C.S.Eng., Surgeon to the Ancoats Hospital, Manchester.

(*Continued from page* 71.)

Naturally, in the early days, the first idea was to kill these organisms by the use of certain drugs, which it was noticed had the power to prevent or greatly to delay their growth. Nobody knew exactly where they came from. They were in the air, on our instruments, our hands, our dress, our tables, our dressings, our patients themselves; and as it was clearly impossible that we could do any operation in a vacuum, and without any of these accessories, attention was first and mainly directed to the finding of some chemical substance which could be depended upon to kill the organisms after they had obtained entrance, which we did not know how to prevent. Carbolic acid, perchloride of mercury, and the various tar preparations, izal, creolin, &c., were apparently the best. Later experience of these showed, however, that they all had the one great drawback that they themselves, though in a far less degree, irritated and injured the tissues we desired to keep unaltered and pure. And if this was true in reference to ordinary tissues, such as skin, muscle, and bone, it was still more intensely accentuated when we had to deal with a delicate absorptive surface, such as that of the peritoneum, the lining membrane of the abdominal cavity.

Luckily, our knowledge has progressed rapidly. We now know—I spare you the names of the discoverers and the details of their experiments—that the air itself, unless it has been lately agitated so as to create dust eddies, is practically harmless, that our great enemy is dust and dirt.

For a moment, let me point out to you that bacteria exist in two forms, the adult bacterium, which alone is able to bring about these changes, but whose life is short and is easily killed, and the spore, or egg, which, whilst it remains so, is powerless, is infinitely minute, is capable of remaining in that state for an indefinite period, is not easily killed, but which when supplied with heat and moisture, develops rapidly into the adult bacterium, passes through a short life, during which it does a definite amount of harm, produces a large number of spores, and dies. The adult bacterium is easily settled. Contact with lotions containing the drugs previously mentioned, exposure to great heat, even continued deprivation of moisture, will kill it. The spores, however, are not so easily disposed of. They settle down contentedly amongst the dust on the floors, on our clothing, on our dressings, on our towels, our blankets, our beds, waiting for the inevitable chance which our copybooks tell us always comes to those who can afford to wait. Worse than all, they settle on our bodies, penetrate into the mouths of the sebaceous glands of our skin, and there, mixed with the greasy material normally present in those tubes, are most difficult of all to dislodge. They get under our finger nails, and there lie perdu amongst the dry epithelium which accumulates there, waiting, ever waiting, until our fingers touch some raw wound, where they find at once the conditions of active life, warmth, moisture, and suitable food.

Abdominal surgery emphasised the necessity of finding some other plan for rendering our patients secure than that of the use of poisonous drugs to the wounded part, and that has been found in two ways. First, it has been found that dry or moist heat alone will sterilise absolutely instruments, dressings, towels, ligatures, sutures, if they are exposed thoroughly to a temperature of 212 deg. for fifteen to twenty minutes. Secondly, we have learned to use these drugs to ourselves, to the unbroken skin of our hands and arms, besides that of our patients, before we touch them, and to use them in such a way as to remove the greasy material and dried epithelium in which our foes lurk. Thirdly, we have learned in many cases to dispense with dressings, and to cover our wounds at once with an impermeable coating through which bacteria cannot enter. But in order to do this we must first be certain that no bacteria or spores are shut up inside, or the results would be disastrous. Now how does all this affect you in the preparation of patients for abdominal section and in your duties during operation?

Septic germs are no respecters of persons. They have no pride. They would just as soon ride into what is to them the luxurious interior of an open abdomen on a sponge as on a ligature or an instrument, and it matters nothing to them whether a nurse is so proud of her general neatness that she thinks extra care at such a time unnecessary, and so leaves them comfortably undisturbed beneath the elegant outline of a filbert-shaped nail until their chance comes, or if she is so self-conscious that she touches up her hair before she hands a sponge, and so carries them from that resting-place to their desired haven, or is so careless that she drops a sterilised ligature and picks it up again, with the invisible but surely present microbe hanging on by his eyelids during the transit. The spore is serenely indifferent, but once in, it gets to work all the same. And so you will see that the patient on the table is really at the mercy of all of us. Three out of four nurses may take all the precautions I am about to mention, the surgeon and his assistant may be as painstaking as possible, the fourth nurse has it in her power by carelessness and indifference to wreck all their work, and it will then be but sheer luck which will decide whether the patient escapes with a good result, has an internal abscess, with months, it may be years, of invalidism, or dies, a victim to pyæmia or septicæmia.

So you will see that it is not mere faddism, or ostentatious fuss, which prompts the methods of preparation I recommend, and that the careful preparation which the surgeon makes of himself should be followed to the smallest detail by all who take part, however small, in the operation. The patient's skin or the lining membrane of her vagina must be first prepared. We begin the day before, because, especially here, the patient's skin, even on the abdomen, is often covered by layers of dead epithelium, which must be soaked and removed before any thorough sterilising can be done. So we give them a hot bath with plenty of soap and water to start with. Then over the abdomen is placed a compress of lint, wet with a solution of some antiseptic, and covered by jacconet, or oiled silk, which is bandaged on. This acts as a poultice, and softens thoroughly the old scurf skin, which in twelve hours can be easily rubbed off, leaving the real skin to act upon. Now, your previous lectures will have taught you that skin is not a flat surface, like that of marble, but contains numberless little pits, the openings into the sebaceous glands which secrete the greasy stuff I have been speaking about, and in which bacteria are very apt to lodge. This must somehow be dislodged, and these tubes cleaned out. Spirit or ether will dissolve this out, especially if you use a flesh brush with it. In order to kill two birds with one stone I usually dissolve biniodide of mercury in this, so that the same action which brushes out the dissolved grease kills the bacteria in it, and leaves the little pit full of some more bacteria poison ready to kill any more spores which may hatch out or drop in. And I depend a good deal upon the mechanical action of the brush, which in private practice is always a new one and in hospital should be fairly new, have not been used for any other kind of work, and should have been soaking in boiling water for a few minutes. When we think the skin thoroughly cleansed, and that is after at least three minutes' scrubbing, we cover it again with a new compress soaked in 1 in 1,000 biniodide solution and keep it on until the patient is on the table. When there it is removed, and a last sponge down is given just before the skin is cut. If the skin should be distinctly dirty turpentine is scrubbed before the spirit, but spirit is always used after it to wash it away or the skin might be blistered and watery solutions alone would have no effect. In such a case it is turpentine first with brush, spirit with brush, then wash with watery biniodide solution.

(*To be continued.*)

### Post-Graduate Clinics for Nurses.

By a Trained Nurse.

### ARTIFICIAL METHODS OF FEEDING.

### Rectal Feeding.—II.

Cases of temporary rectal feeding—that is to say, those in which the necessity arises from a hæmatemesis, or the presence of a foreign body in the œsophagus, or a temporary inflammation of mouth, pharynx, or œsophagus—do not present the same difficulties as do those in which rectal feeding may represent all the nourishment a patient is able to take for many days or weeks. The nurse must bear in mind that such a patient soon runs down below par, and that it is her province to prevent this.

Rectal feeding is often ordered in the first instance because of the exhausted condition of the patient, who by long and severe fever or serious gastritis or gastric cancer has been living on low diet for some length of time. The objection to this method of feeding on the part of some patients—amounting in many cases to a real repugnance—very often leads to the postponement of rectal feeding until they are already very weakened. Frequently a nurse needs much tact and persuasion to induce her patient to consent to nutrient enemata. In all cases the nurse needs delicacy in this method of feeding her patient. And there is no doubt that the temperament — the wholesome healthy matter-of-course demeanour of the nurse—has much to do with the influencing of the patient in a question of this kind. Nurses who are strongly imbued with the sense that each process of the body is equally refined and wholesome, that every part of the human body is a beautiful and wondrous piece of mechanism, soon induce their patients to feel in an equally wholesome manner.

When nutrient enemata are first resorted to the nurse must try to disabuse her mind of the conviction that the bowel will soon, and necessarily, become so irritable as to reject the injected nutriment. Given with a short hard syringe, the material of which is most liable to rub against and irritate the folds of a delicate mucous membrane, the bowel may easily become most irritable. But such syringes should not be used. It is common enough for a nurse to begin with the syringe, and then when the bowel shows signs of irritability and rejection of the enema, to substitute a flexible catheter. But this is the old story of shutting the stable door when the steed has vanished. For once establish rectal irritability, and the slightest stimulus will carry it on. I have seen nurses administering nutrient enemata with a steel-nozzled syringe or one of the hardest rubber kind, and these same nurses were loud in their belief that nutrient enemata were of little value. Given in that way doubtless they are, but the clever and well-instructed nurse would scorn such a primitive method. The first point of importance is that the rectum—so long as nutrient enemata are being given—should be kept free of fæces and mucus. In normal cases the rectum should be empty, for the accumulation of fæces occurs higher up in the bowel. In other cases the rectum may be packed, and it is necessary as a matter of routine to wash out the bowel as the initial step to rectal feeding and to keep this up daily, using a two to three pint solution of soapsuds and giving a nutrient enema almost immediately after.

In cases where obstinate constipation has existed or impacted fæces are present, an enema given in the knee-chest position proves most efficacious. A soft flexible rubber cathether should be selected—not too soft, however, or it may double back—and the first principle the nurse must fix in her mind is that the tube must pass up eight or nine inches before the fluid is injected. Nutrient enemata are very commonly injected two or three inches above the anus; the greater part returns, anal irritation soon supervenes, and the nurse "doesn't believe much in nutritive enemata." In addition to the danger of a speedy return of the injection, it must be remembered that given thus the food does not spread over the large absorbing surface, as happens when it is injected high up.

Olive oil should be used for the catheter—glycerine never—for, as is well known, this is most liable to set up peristaltic action. A little olive oil should be applied also to the anus, and the catheter should be warmed. A small syringe capable of holding two or three ounces should be used with the catheter, and care taken to expel all the air before connecting it with the catheter. And it is well to connect the syringe with the catheter and fill the tube with fluid before passing it into the rectum, slightly pinching the tube, for no one factor interferes so much with the success of nutrient enemata as does the presence of air in syringe or tube. This, naturally enough, sets up peristalsis and excites the bowels to move.

The introduction of air with food doubtless has much to do with the production of the so-called "irritability of the bowel." The patient should lie on the left side with the hips raised high by means of packed pillows, and above all things, the introduction of the catheter must be slow, gentle, and without fuss or excitement. Mental emotions soon react on the intestinal tract, and any hurry may result in such nervousness in the patient that the injection may be rapidly returned. I have seen most wonderful results from the giving of nutrient enemata to patients placed in the "Sims" position—with the hips more raised, however, than when this posture is assumed for gynæcological purposes. A firm pad pressed against the anus for 15 to 20 minutes after the tube is withdrawn is a time-honoured and always excellent method to adopt. I omitted, when speaking of the daily enemata for the purpose of washing out the bowel, to remind the nurse that these, too, should be given by means of a catheter. The ordinary tube is too liable to cause some irritability to the mucous membrane to risk using it in a case where it is so important to avoid any likelihood of this. By using the catheter—and the calibre of this depends on the age of the patient and the condition of the bowel—for both purgative and nutritive purposes the nurse at least gives her patient the very best chance of obtaining the benefit of rectal feeding. Given in the way I have described I have seen patients suffering even from hæmorrhoids—and these the nurse knows prove a serious stumbling-block to nutrient enemata—retain and receive the benefit of food injections for a couple of weeks before diarrhœa showed itself. It may sometimes happen even when all the conditions are as carefully attended to as those just set forth for the nurse's guidance that the first half-dozen injections may be returned, but if the nurse continue, with every precaution ingenuity can suggest, giving only om two to three ounces at a time, and taking care that the temperature of the fluid when it reaches the bowel is from F. 92 deg. to F. 96 deg., the bowel may get used to the process and become immune, as it were. An injection is a foreign body, and the healthy bowel is only doing its duty in attempting to expel it. But the mental attitude of the patient, the knowledge on his part that it is advantageous to him to retain it, makes all the difference, and may set up a counteracting influence to the natural instinct of expulsion. I particularly said in speaking of the temperature of the injection that this should *reach* the bowel at the given degree of heat, which may be practically assured by allowing for the loss of three to four degrees in the giving. It must not be forgotten that the more the injection approximates the normal heat of the intestines the less it is like a foreign body. Given too hot or too cold it acts as a stimulus or excitant to peristaltic action, and an unfavourable element is thus introduced.

### Nursing in Paris Hospitals.

### B.—THE TRAINING OF NURSES.

V.—The Schools for Midwives: La Maternité.

Paris possesses two Government schools of midwifery, one established by Napoleon in 1810, the other only some nineteen years since under the Third Republic. The first is located under the picturesque gables of the historic old Port Royal convent. That pride of Southern Paris, dear to the neighbouring art schools of Montparnasse, has long sheltered the famous lying-in hospital, La Maternité. The hospital and school are, however, quite separate administrations, though the hospital patients are the charges of the one, and the material for the other.

Strange as it may seem, neither have the ordinary nurses in La Maternité any special qualifications required of them nor is any advantage taken of the exceptional opportunities to give them any special training in the treatment of childbirth. Of course, the births themselves are under the charge of qualified midwives, but otherwise the patients, both mothers who are admitted a month beforehand, and mothers and children who are allowed to remain for a short period after and in case of serious illness for a considerable period, are in charge of ordinary nurses.

The Maternité School, although under the control of the Paris Assistance Publique, is designed for the whole of France, having only paying students, some at their own expense, and many holding scholarships from the different French departments, or, in some cases, from the local commune or hospital administration.

The school year is from July 1st to June 30th, scholars being admitted each year during the whole of June by examination, if a school certificate is not produced; for the midwives, unlike the ordinary nurses, are expected to have some preliminary education. Scholars must be between 18 and 35 years of age at admission. They must be provided with various other certificates besides the school one, including one of good conduct from the mayor of their commune, account of their relatives, of revaccination within two years, and in case of marriage, the consent of the husband and also his civil description.

Scholars have all to pass a medical examination by the doctor of La Maternité, to see that they are physically qualified for midwives, and no pregnant woman is admitted.

The school fee is 1,000 francs per year, payable quarterly in advance. At entrance the total amount to pay is 302 francs; *i e.*, 250 francs for the first quarter, 42 francs for books, and 10 francs for instruments considered indispensible for all students. The scholars holding departmental scholarships, however, have only to pay for the instruments, the rest being arranged in the Government accounts.

The scholars are lodged, fed, and furnished with bed and table linen and aprons. They are allowed three francs a month for washing. No special outfit is required to be provided by them. They have a uniform of black with blue trimmed body for the first year, and red for the second year.

In case of illness, the scholars are attended gratuitously by the Maternité doctors. They are only allowed to have but one outing a month from the hospital during the whole course, and then only with their father, mother, or husband, or under the care of some person appointed by their friends or the administration.

The course lasts two years, including about 120 lessons each year, given either by the chief or the assistant chief accoucheur, and a few lessons by the hospital physicians.

When I visited the Maternité school this year there were 90 students, but there is accommodation for 114. Beside this places are found for about 12 more students in the other hospitals.

During the months of May and June the annual examinations take place, the examining board consisting (with an accoucheur delegated by the administration of the Assistance Publique) of a professor of the Faculty of Medicine, the chief accoucheur, the assistant chief accoucheur, and a physician of the hospital. Each of the last three mentioned chooses a subject for the written composition, and the subject chosen is drawn by lot from these three. About three hours is allowed for the composition. The oral examination lasts from 15 to 20 minutes.

The general examinations are complicated with several series of special examinations for different prizes. Thus, in 1897, the general examination was concluded on May 21st, having proceeded on six previous dates. The subject of the composition having been given, the 38 pupils, who had all followed the courses for two years, had their compositions presented in sealed envelopes, which were opened and examined. All the 38 were declared sufficiently qualified, on payment of 25 francs, to receive a diploma entitling them to exercise the profession of midwife throughout France. Nevertheless, 25 of the 38 were selected as worthy to contest for the gold medal, a contest consisting of three additional examinations, in two batches of 13 and 12 each at the first two, and a further selection of 12 out of the two batches of the third examination. At all three, different subjects of composition were given. Finally, on the third day, the gold medallist—in this case Mdlle. Louise Grasset, of Versailles—was chosen. Mdlle. Grasset, who seems to have been a sort of female Admirable Crichton in midwifery, made a neat valedictory speech on behalf of her colleagues, acknowledging their loyalty to the great traditions of La Maternité of Paris, which, with the reply of the head examiner, is printed in the annual report. Numerous minor prizes are given at the general examination, while other examinations for special prizes in anatomy, physiology, hygiene, pathology, &c., take place, the chief of which prizes in 1897 seem to have been carried off by the gold medallist.

In concluding this account of the Maternité School, a few totals of the year's work during the last completed school year may be of interest. The total number of accouchements was 2,547, of which 2,402 were natural, 41 false presentation of the infant, 90 delivered with the forceps, 9 by perforation or cephalotribe, 1 by embryotomy, and 4 by cæsarian section. The children consisted of 1,982 single and 14 twins (living) at proper term, 285 single and 49 twins (living) premature, or 2,330 living in all, while 217 were born dead, being 18 at term and 83 premature still births, and 56 at term and 60 premature deaths at birth. There were 149 miscarriages, 18 cases of eclampsy, 5 of anencephalia, 67 cases of hæmorrhage, 46 syphilitics, 28 of placental presentation, 40 umbilical cord protrudance, and 1 each of seccrophaly, symphysiotomy, and of miscarriage of twins.

Edmund R. Spearman.

### Bishop Auckland and District Nursing Association.

SUCH a report as that just issued for the tenth successive year by the Bishop Auckland and District Nursing Association brings the enormous value of the district nurse strongly home to its readers. Here is a record of untiring work amongst the homes of the poor, work which means more than merely the alleviation of suffering, for with it is spread abroad knowledge on essentials of health and cleanliness.

The association has nearly doubled its work in the last seven years, the number of cases nursed last year (1897) being 342, as against 186 in 1892. The figures are:—

| Year. | Patients. | Visits Paid. | No. of Nurses. |
|---|---|---|---|
| 1892 | 186 | 4,588 | 2 |
| 1893 | 274 | 7,789 | 2 |
| 1894 | 262 | 8,413 | 2 |
| 1895 | 315 | 8,492 | 2 |
| 1896 | 310 | 7,921 | 2 |
| 1897 | 342 | 8,159 | 2 |

The association is in affiliation with the Queen Victoria's Jubilee Institute for Nurses, and during the year the nurses' work was inspected by Miss Armstrong and very satisfactorily reported on. The detailed subscription list at the end of the last report shows that those who benefit by the services of the nurses appreciate them to some extent, £120 16s. 3d. being contributed by collections from the town districts. Nevertheless, the committee consider that the obligation is hardly sufficiently recognised, and they point out that the income from these legitimate sources was short of the outgoings by nearly £30, the deficiency being made up by proceeds from Lady Eden's entertainment and a charity ball. Thanks to this help a balance in hand of £32 15s. was reported at the end of year, the total expenditure having amounted to £211 8s. 4d. Offertories amounted to £17 8s., and workshop collections to £12 18s. 9d. The Guardians of Bishop Auckland very properly contribute £10, seeing that a good many of the patients visitedare on parish relief.

Last year it was decided to commemorate the Diamond Jubilee by the establishment of a nurses' home. Owing to the many calls on the public purse at the time this scheme had to be deferred, but it is now very desirable to make an effort to obtain the necessary funds if the commemoration plan is not to be abandoned. Subscriptions already promised amount to £202 8s., the sum aimed at being £800. At the annual meeting held in March it was decided that a reserve of £205 should be transferred to a fund for the "Home," while the donors of a further sum of £20, originally collected for a hospital in connection with the association, should be asked to consent to its addition to the fund. It is very much to be hoped that the home will soon be an accomplished fact, and that the matter will be taken up whole-heartedly by the people of Bishop Auckland and its neighbourhood. Sir William and Lady Eden set an excellent example, and the members of the committee take a great personal interest in the association. If this spirit spreads as it should do there ought to be little difficulty in raising the necessary money, both for the home and also for the support of a third nurse, for whom there is plenty of work. Dr. Wardle laid emphasis on this fact at the annual meeting, when he spoke warmly of the excellent work of the superintendent nurse, Miss Violet E. Wood, and her assistant, Miss Fisher.

Lately the association has acquired that district nursing necessity, a bicycle, thanks to the energy of Mr. Wild and Miss Wood, and the nurses find their new possession very useful.

The arrangement of the work is left, as is enacted in the bye-laws, in the hands of the superintendent nurse, who is responsible for the nursing. The bye-laws also provide that the nurses' working day shall not exceed eight hours, except under special circumstances. Such "special circumstances" must have arisen pretty frequently of late, one may suppose, looking at the great number of cases visited by the two nurses last year, and it is clear that a third nurse must be added to the staff if the present willing workers are not to have more on their shoulders than they can properly undertake, with justice to themselves and their patients. The administration of the association, subject to the control of the members, is vested in an executive committee of ten ladies and ten gentlemen, with a secretary and treasurer. The office of chairman to this committee is at present held by Mr. Joseph Lingford. The association is under the patronage of the Bishop of Durham, and the president is Sir William Eden.

### Our American Letter.

IT has been recently estimated that there are about 15,000 nurses in America, and nearly half of that number are to be found in the neighbourhood of New York and Brooklyn. Every effort is being made by the leaders amongst them to induce them to combine to safeguard both their professional and social privileges. Mrs. Willard, the founder of the Metropolitan Club and Registry for Trained Nurses in New York City, addressed the members of the Associated Alumnae Registry for Nurses very forcibly upon the subject. "Organisation means strength; it is also an educator," she reminded her hearers. "You can better fight for your profession together than you can singly; you can make it nobler, better in every sense of the word."

The guild of St. Barnabas Club have taken the upper floor in addition to their present premises. The members have recently arranged what they have named a "crib club," and on Saturday afternoons during the tea hour those present ply needle and scissors, fashioning garments for such babies as arrive without due provision having been made for them, the maternity nurse employed amongst the poor distributing them as needed. Five hundred women, trained and untrained, have offered themselves as Red Cross Nurses at the hospital in New York. Of these, 200 have been provisionally accepted. One and all are required to take the following oath, and some very impressive scenes have taken place lately during its administration:—

"I hereby solemnly and sincerely promise and declare that in entering the New York Red Cross I will abide by its rules and regulations in every particular. I will serve the Red Cross as required, and faithfully perform every duty prescribed or ordered for me when directed by my superior officers. I will prepare all articles required by the sick and wounded of mankind with utmost care and conscientiousness, and should I ever, through unintentional oversight, make an error, or see it made, I faithfully promise to report the same without hesitation, that no misery in the life of any being may occur through my oversight or error. Furthermore do I promise that I shall act as a true sister to every human being, independent of nationality, creed, or sex; that there should be nothing too small and nothing too great an effort for me to assist in relieving pain, if within my power so to do, and if within the limits of Red Cross work. Furthermore do I promise that I shall act as a true sister to every person associated with me in the Red Cross, recognised by the officers of the American National Red Cross; that I shall hold sacred the emblem and uniform of the Red Cross, and shall never use or wear them except under the direction of my superior officers of the Red Cross Hospital, authorised by the American National Red Cross. Furthermore do I promise allegiance to my sister-in-chief, to the president and officers of the New York Red Cross Hospital, and president and officers of the National Red Cross. All of which I solemnly and sincerely promise in the presence of God, the officers and members of the New York Red Cross Hospital, and to which I pledge my sacred honour."

### A Book and its Story.

### THE KING WITH TWO FACES.

MISS COLERIDGE's historical romance* deals with the life and times of Gustavus the Third, the Swedish king, whose striking characteristics of great personal bravery and humanity, combined with an enigmatical magnetic temperament, made him difficult of comprehension, even to the most devoted of his followers. He ascended the throne in 1771 at a period when men of character and distinction were numerous amongst his contemporaries. Whether the surname by which he is known was due alone to an accident of birth, or to a rapid change of front to suit the occasion, we leave to the readers of this brilliantly written romance to find out. Its setting is Sweden. Towards its close the French Revolution is raging. At home Gustavus, beloved by the peasants, whose lot he had done so much to alleviate—" Was it not the King who had made the desert to blossom as a rose?"—was harassed by invading enemies without, and the hatred of the nobles, whose dissensions within were even more dangerous to the country's peace.

The story opens on a striking scene in which three conspirators await the arrival of a king's messenger, the young Count Adolph Ridding. Out of the darkness of the night through which he is riding he comes upon the open door of the house. Hastily dismounting, he enters, to find himself in a barred room from which he can only escape by revealing the message, only to be delivered by word of mouth, to the governor of the beleaguered city of Gothenburg. To turn traitor is impossible. After various expedients were tried to make his escape, Ridding resorts to a final one by working on the credulity of the chief conspirator, Auckarström. He judges from his wearing a charm against the Evil Eye that he is susceptible to occult superstition, and relates rapidly a recent prophecy of the notorious Sybil, Moorna Afridson, that it was by his — Ridding's — hand that the King should die. "Whilst uncertain of his fate, Adolph watched intently, though without the intolerable sensation of suspense which had before oppressed him. He had done something. The weight of utter hopelessness was gone. . . . Some kind of change had come over the leader. . . . The rest were holding their breath. All about the room the shadows seemed to grow thicker." At last a voice spoke out of the darkness: "I war with the King, not with the King of kings. Young man, go free."

To deliver the King's message at Gothenburg was only part of his mission. Ridding had also to arouse the Northerners for the King's defence. Mora was the rallying point, and the signal for rising was to be the lighting of beacons on the hill tops. He continued his journey, and his next halt is made at the castle of his uncle Carl de Greer. A ball is being given, and his sudden appearance causes more surprise than pleasure to all who, like de Greer, are opposed to the King. Adolph's lovely cousin, to whom he is secretly betrothed, shows alone delight. She, by some intuitive faculty suitable to the situation, had felt that he was near. There is a stormy exchange of sentiments on the part of de Greer and a Baron Essen with Adolph, and the Messenger once more starts on, taking refuge in a neighbouring hut on "Reindeer's Crag," in response to Tala's parting injunction to meet him there. She comes later with food and refreshment, and a charming chapter is devoted to their meeting, the delicate, half-serious badinage of the lovers breaking in brightly in strong relief on the sombre environment of the scene and the great issues at stake, while Tala moves a ministering angel in the foreground of the picture. They had lighted a fire in the hut, which becomes ignited; the flames raged and crackled in the darkness, and so the first beacon was lighted by the lovers. "Glorious! glorious! Everyone will see it. The country will be up in arms to-morrow!" "He got no answer. . . . She stood there cold and white in the fierce red gleam of the flames. She took the torch and reversed it. That was his last vision of her; in the moment that came after she was too close for any vision." Ridding reaches Mora, finding *en route* the people everywhere enthusiastic for the royal cause. The King, disguised as a countryman, had reached Mora already, and was addressing a large crowd of rough Dalecartians. Ridding hears a voice exclaiming, "The King has the face of an angel; ay, the face of an angel, but he has two faces, you know." And for the first time Ridding notices that the two sides of the face do not correspond. At Gothenburg the King makes a brave stand and saves the city, Adolph at the risk of his life fires a mine and destroys the bridge at the King's command. Afterwards he is despatched to Paris. He returns, after an absence of three years, to find a *coup d'état*. His beloved King, disloyal to himself and Tala, lying at the point of death, married to his rival. It is only by her entreaty that he is restrained from firing the fatal shot which ends the King's life. He dies by the hand of the assassin Auckarström at a masquerade.

It is impossible to form from the above slight outline an idea of the charm and lucidity of Miss Coleridge's style. Historical romance is too often dull reading, but fancy is here blended with fact in so fascinating a manner that the result is a story of unflagging interest from first to last.

* "The King With Two Faces." By M. E. Coleridge. (London Edward Arnold). 6s.

### Appointments.

### MATRONS.

ROYAL HOSPITAL FOR INCURABLES, DONNYBROOK, DUBLIN.—Miss Louisa Bradshaw was appointed Lady Superintendent of this hospital on May 26th, 1898. She was trained at the Adelaide Hospital, Dublin, and then became night superintendent, and afterwards assistant matron, of the General Hospital, Nottingham.

### Minor Appointments.

BELFAST ROYAL HOSPITAL.—Miss Agnes Green, the recently-appointed Night Superintendent of the Belfast Royal Hospital, was trained at the Children's Hospital, Edinburgh, and at the Western Infirmary, Glasgow. She was at one time sister at the National Orthopædic Hospital, London.

CORK STREET FEVER HOSPITAL AND HOUSE OF RECOVERY, DUBLIN.—Miss Lilla Dawson, who was trained at University College Hospital, London, and who was afterwards matron of the Bromwich Hospital, Birmingham, has been elected Assistant Lady Superintendent and Matron of this hospital.

EAR AND THROAT HOSPITAL, BIRMINGHAM.—Miss Edith Hanlock was appointed Sister of this hospital on May 4th. She was trained at St. Bartholomew's Hospital, London, and at the Derbyshire Royal Infirmary. She has recently been engaged in private work.

DEVONSHIRE HOSPITAL, BUXTON.—Miss Sarah Horsley, who received her training at Oldham Infirmary, was appointed Head Nurse here on June 1st. She has held the position of staff nurse at the Hartlepool and the Peterborough Hospitals.

TONBRIDGE WORKHOUSE.—Miss Maggie Evans, for four years charge-nurse at St. John's Infirmary, Hampstead, N.W., has been appointed Superintendent Nurse of this workhouse. She was trained at the Central London Sick Asylum

WINCHCOMBE UNION WORKHOUSE.—Miss E. Green was appointed, on May 21st, Assistant Matron and Nurse of the above workhouse. She was trained at Hackney Union Infirmary, Homerton, London, N.E.

### For Reading to the Sick.

THE SEASON OF TRINITY.

"HOLY, Holy, Holy, Lord God Almighty, which was, and is, and is to come."—*Revelations* iv. 8.

**Verses.**

Three in One, and One in Three,
Ruler of the earth and sea,
Hear us while we lift to Thee
Holy chant and psalm.

Three in One, and One in Three,
Dimly here we worship Thee;
With the Saints, hereafter we
Hope to bear the palm.
—*Dr. G. Rorison.*

Once in His Name Who made thee,
Once in His Name Who died for thee,
Once in His Name Who lives to aid thee,
We plunge thee in Love's boundless sea.
Upon thy death-bed name it,
So mayst thou chase the infernal horde,
So learn with Angels to proclaim it,
Thrice Holy, one Almighty Lord.
—*John Keble.*

O Unity of Threefold Light,
Send out Thy loveliest ray,
And scatter our transgressions' night,
And turn it into day.
Make us those temples, pure and fair,
Thy glory loveth well,
Thy spotless tabernacles, where
Thou mayst vouchsafe to dwell.
—*Eastern Hymn.*

Holy! Holy! Holy! though the darkness hide Thee,
Though the eye of sinful man Thy glory may not see;
Only Thou art holy, there is none beside Thee,
Perfect in power, in love, and purity. —*Heber.*

Are we not holy? Do not start!
It is God's sacred will
To call us temples set apart
His Holy Ghost may fill. —*A. Procter.*

**Reading.**

Behold, a door was opened in heaven . . . and, behold, a throne was set in heaven . . . and there were seven lamps of fire burning before the throne, which are the seven spirits of God.—*Revelation* iv. 1, 2, 5.

Baptism in the name of the Three Persons in One God is as the door opened in heaven. To us, as born of water and of the Spirit, are the mysteries of heaven made known, which eye hath not seen nor ear heard. In other words, to us a door is opened in heaven, and the mystery of the God-head is reflected in the sea of glass which is before the throne. At the first creation the Spirit moved on the face of the waters, even now so it is in the Christian kingdom. The seven lamps and the sea of glass are the description of Christ's kingdom after the day of Pentecost, explained to us by earthly similitudes. For who is equal to these things? And who should understand them if God did not come down to us in our weakness, and meet us in our infirmities, over-coming by his humility our pride?—*Isaac Williams.*

### Where to Go.

QUEEN'S HALL.—On June 11th the pupils of the North Hackney High School will give a demonstration of physical exercises to raise funds for the London School Nurses' Society. This society, when established, will supply visiting nurses for the elementary schools in the poorer neighbourhoods of London. Tickets may be obtained from Miss Honnor Morten, Ivy Hall, Richmond.

BY the kindness of the family of the late Sir John Millais, the summer sale of the Working Ladies' Guild will be held at 2, Palace Gate, W., on June 8th, 9th, and 10th. The Duchess of Albany will open the sale on Wednesday, June 8th, at noon. The hours on the following days will be from two to seven p.m.

### Notes and Queries.

**The contents of the Editor's Letter-box have now reached such unwieldy proportions that it has become necessary to establish a hard and fast rule regarding Answers to Correspondents. In future, all questions requiring replies will continue to be answered in this column without any fee. If an answer is required by letter, a fee of half-a-crown must be enclosed with the note containing the enquiry. We are always pleased to help our numerous correspondents to the fullest extent, and we can trust them to sympathise in the overwhelming amount of writing which makes the new rules a necessity. Every communication must be accompanied by the writer's name and address, otherwise it will receive no attention.**

*Lady Roberts' Nurses.*

(87) Miss Paton would be glad to know where she could get information with regard to Lady Roberts' Nurses for India.

The aim of the fund which bears Lady Roberts' name is to supply homes in the hills for the nurses working in the military hospitals. (2) To provide officers' hospitals. (3) To provide auxiliary nurses to hospitals where the Government has not done so. Lady Roberts selects this auxiliary staff from nurses personally known to her, consequently no outside applications for admission are entertained.

*A Holiday Home.*

(88) Can you tell me of a cheap holiday home where a nurse who has been ordered three or four months' complete rest could be received at reduced charges?—*M. E.*

Apply Hon. Secretary, Lady Smyth's Home, Long Ashton, near Clifton. For tired workers. Charges 6s. and 8s. 6d. a-week. Apply Mrs. Theodore Mander, Wightwick Manor Farm, Wolverhampton. Apply Lady Superintendent, Convalescent Home for Women and Children, New Brighton, Cheshire. Terms from 6s. 6d. to 21s. weekly.

*Diet for the Diabetic.*

(89) Will you kindly give me a list of diet with plenty of changes for diabetic patients?—*Miss C. G.*

You might consult Mrs. Ernest Hart's "Diet in Sickness and in Health" (price 3s. 6d.) Miss Maude Earle has devoted a special section to this class of diet in her new cookery book. The Scientific Press, London, will supply either book.

*Invalid Homes.*

(90) Will you tell me if there are any convalescent homes on the East Coast where patients requiring assistance in the toilet are received?—*S. E. A.*

There are a number. Consult list in "Burdett's Hospitals and Charities." You could see a copy at this office if you have not one.

*A Day's Work.*

(91) Can you tell me what London and provincial hospitals have an eight or nine hours' day's work extending to non-paying as well as to paying probationers as at Guy's?—*Guide.*

There are very few, as the Poplar, the London, King's College, and Guy's Hospitals may be said to have inaugurated the movement demanding shorter hours for nurses.

*List of Hospitals.*

(92) Kindly give me the names of several good hospitals, including two or three children's hospitals, where the premium for probationers is not high.—*E. E. G.*

Consult Honnor Morten's "How to Become a Nurse," price 2s. 6d., from the Scientific Press. In it you will find full particulars of all the training schools.

*Medico-Psychological Association.*

(93) Can you tell me how I can obtain the medal of the Medico-Psychological Association?—*Ralph.*

Write to the Registrar of the Medico-Psychological Association, 11, Chandos Street, W., for particulars.

*Apothecaries' Hall Examination.*

(94) Can you tell me of a good lady "coach" for the Apothecaries' Hall examination?—*S. L.*

Miss Lamport, 52, St. John's Wood Road, N.W.

*Rules for a Parish Nurse.*

(95) Kindly tell me where to get information as to the best rules for a parish nurse and her maintenance. Under what circumstances can a grant be obtained from the Board of Guardians, the District Council, or both?—*W.*

Could you tell me where I could get information as to the conduct of district nursing associations, advice for the committees, and if it is customary to move nurses from one district to another?—*Beta.*

Chapter VIII. in H. Morten's "How to Become a Nurse" is upon this subject, and we published a letter on "How to Start a District Nurse" in the "Mirror" for March 26th, which gives some useful hints. Success depends so much upon local conditions, that the secretary of the nearest county associations would probably be able to give the most practical advice for your district. Grants are permitted from the Guard ans when the district nurse attends out-door paupers, and application for such should be laid before the board.

*Nursing in Cairo.*

(96) "H. O. F." answering "Ramlek's" query, says that she thinks there is a private nursing staff of three nurses in connection with the Kasr-el-Aini Hospital at Cairo, of which Dr. Sandwith is the secretary. There is also a private nursing home in the town kept by Miss James.

*The Nurses' Co-operation.*

(97) I am anxious for private nursing in or near London. Is the Nurses' Co-operation the best association I can join? Please give address.—*Ylil.*

There is none better. Address the Secretary, The Nurses' Co-operation, 8, New Cavendish Street, Portland Place, W.

